# Partially Protective Immunity Induced by a 20 kDa Protein Secreted by *Trichinella spiralis* Stichocytes

**DOI:** 10.1371/journal.pone.0136189

**Published:** 2015-08-19

**Authors:** Kuo Bi, Jing Yang, Lei Wang, Yuan Gu, Bin Zhan, Xinping Zhu

**Affiliations:** 1 Department of Parasitology, School of Basic Medical Sciences, Capital Medical University, Beijing, PR China; 2 Beijing Friendship Hospital, Capital Medical University, Beijing, PR China; 3 Department of Pediatrics, National School of Tropical Medicine, Baylor College of Medicine, Houston, Texas, United States of America; Universidade Federal de Minas Gerais, BRAZIL

## Abstract

**Background:**

*Trichinella spiralis* infection induces protective immunity against re-infection in animal models. Identification of the antigens eliciting acquired immunity during infection is important for vaccine development against *Trichinella* infection and immunodiagnosis.

**Methods and Findings:**

The *T*. *spiralis* adult cDNA library was immunoscreened with sera from pigs experimentally infected with 20,000 infective *T*. *spiralis* larvae. Total 43 positive clones encoding for 28 proteins were identified; one of the immunodominant proteins was 20 kDa *Ts*-ES-1 secreted by *Trichinella* stichocytes and existing in the excretory/secretory (ES) products of *T*. *spiralis* adult and muscle larval worms. *Ts-*ES-1 contains 172 amino acids with a typical signal peptide in the first 20 amino acids. The expression of *Ts*-ES-1 was detected in both the adult and muscle larval stages at the mRNA and protein expression levels. Mice immunized with recombinant *Ts*-ES-1 (r*Ts*-ES-1) formulated with ISA50v2 adjuvant exhibited a significant worm reduction in both the adult worm (27%) and muscle larvae burden (42.1%) after a challenge with *T*. *spiralis* compared to the adjuvant control group (*p*<0.01). The r*Ts*-ES-1-induced protection was associated with a high level of specific anti-*Ts*-ES-1 IgG antibodies and a Th1/Th2 mixed immune response.

**Conclusion:**

The newly identified r*Ts*-ES-1 is an immunodominant protein secreted by *Trichinella* stichocytes during natural infection and enables to the induction of partial protective immunity in vaccinated mice against *Trichinella* infection. Therefore, r*Ts*-ES-1 is a potential candidate for vaccine development against trichinellosis.

## Introduction

*Trichinella spiralis* is a tissue-dwelling nematode that infects a wide variety of vertebrate hosts including humans, in most areas of the world [1,2]. Human infection occurs by eating raw or undercooked meat containing infective *Trichinella spiralis* larvae [3]. Because of changes in diet and cooking practices and an increase in the consumption of meat, trichinellosis caused by *T*. *spiralis* infection is regarded as an emerging or re-emerging infectious disease [4,5]. It has been estimated that more than 11 million people could be infected in the world [6]. Outbreaks of trichinellosis in humans have been regularly reported in different areas of the world [6,7]. This zoonosis is both a public health challenge and an economic issue in porcine animal production and food safety [8,9]. Therefore, the development of vaccines against *Trichinella* infection in livestock and humans is needed as an effective approach to control this disease.

All of the developmental stages in the life-cycle of *T*. *spiralis* occur in the same host, including the adult worm in the small intestine and the larval stage that develops in the muscle to form cysts [10]. Protective immunity induced by primary *T*. *spiralis* infection has been observed in different infected animals [11–13]. Infection-induced resistance to secondary infection is related to a potent Th2 response and high antibody titer [11,12]. However, the complete mechanism of protective immunity and which antigens induce protective immunity in the host remain unknown. Therefore, identification of the antigens produced by *T*. *spiralis* that elicit host protective immunity is critical for understanding the protective mechanism and targeting these antigens for vaccine or drug development for the control of trichinellosis.

To identify the protective antigens during infection, the adult cDNA library of *T*. *spiralis* was immunoscreened with *T*. *spiralis*-infected swine sera. More than forty positive clones were recognized by the *T*. *spiralis*-infected sera, and one 20-kDa protein secreted by *T*. *spiralis* muscle larvae and adult worms was cloned and characterized. Significant protection was induced in immunized mice against *T*. *spiralis* infection. Here, we describe the screening, molecular characterization and evaluation of the protective efficacy against *Trichinella* infection induced by this antigen in a murine model.

## Materials and Methods

### Parasites and antigen preparation

*T*. *spiralis* (ISS533) was maintained in female ICR mice. Muscle larvae (ML) were recovered from infected mice using a modified pepsin-hydrochloric acid digestion method as previously described [14]. Adult worms were collected from the intestines of infected mice four days following larval challenge. Mice were euthanized prior to these procedures for collection of parasite. Crude somatic extracts of ML and adult worms were prepared with conventional homogenizing methods [11]. The excretory-secretory products of ML (MES) were prepared and collected as previously described [15]. Briefly, freshly collected *T*. *spiralis* ML were washed three times with phosphate-buffered saline (PBS) and then incubated in RPMI-1640 medium supplemented with 100 U/ml penicillin, 100 U/ml streptomycin and 0.1% bile bovine (Sigma,USA) at 37°C and 5% CO2 for 48 hours. The culture supernatant was collected by centrifugation and was filtered through a 0.45-micron syringe filter and buffer exchanged into PBS. The excretory-secretory products of adult worms (AES) were obtained with the same method as MES except for absence of bile bovine stimulation [16]. The protein concentrations of the prepared worm antigens were determined using a BCA assay (Pierce, USA).

### Animals

Female BALB/c mice aged 6–8 weeks and free of specific pathogens were obtained from the Laboratory Animal Services Center of Capital Medical University (Beijing, China). The mice were maintained under specific pathogen-free condition with suitable humidity and temperature.

All experimental procedures were approved by the Capital Medical University Animal Care and Use Committee and comply with the NIH Guidelines for the Care and Use of Laboratory Animals.

### Sera preparation

*T*. *spiralis*-infected rabbit sera were obtained from two New Zealand white rabbits orally infected with 4,000 *T*. *spiralis* muscle larvae and euthanized with exsanguination after being anaesthetized with 25 mg/kg of Ketamine. Infected swine sera were obtained from four Wuzhishan pigs each orally infected with 20,000 *T*. *spiralis* ML and then euthanized with exsanguination after being anaesthetized with 25 mg/kg of Ketamine. Infected mice sera were obtained from BALB/c mice orally infected with 500 *T*. *spiralis* ML and euthanized with CO_2_ inhalation using methods described [17]. Some of mice showed some weight loss and rough hair coat, but all tolerated for the challenge. Animals were monitored by research personnel every day for general appearance, hunched posture, rough haircoat, labored breathing, lethargy, lameness, ataxia, diarrhea, abnormal vocalization and abnormal discharge from the eyes or nose. If any animals have bleeding diarrhea, labored breathing, severe leg injuries or have become moribund they will be euthanized immediately by CO_2_ inhalation. All infected sera were collected 45 days post infection (dpi) and pooled. All human sera were collected from patients with agreement to donate sera for diagnostic and research purpose. All procedures were approved by the Capital Medical University Animal Care and Use Committee (approval number: 2012-X-108). Infected human sera were collected from *T*. *spiralis-*infected patients living in the endemic area of Yunnan Province of China during an outbreak and were confirmed by positive serological examination and typical clinical symptoms after being excluded from other parasite infections through fecal or blood examination. All human blood samples were collected for routine care and epidemiological investigation and not for the purposes of this specific study, without revealing any identity of patients, according to the protocol approved by the Institutional Review Board (IRB) of Capital Medical University.

### Immunoscreening the adult cDNA library of *T*. *spiralis*

The *T*. *spiralis* adult (5-day old) λZAPⅡcDNA expression library was immunoscreened with *T*. *spiralis-*infected swine sera according to conventional methods. Briefly, 5x10^4^ recombinant plaques on each petridish were incubated at 37°C with IPTG-soaked nitrocellulose membrane (NC) discs (Amersham Biosciences, UK) overnight. Each membrane disc was then blocked with 5% dry milk-PBST (PBS+0.05% Tween 20) overnight at 4°C and subsequently probed with pooled sera from pigs infected with *T*. *spiralis* (1:10,000) for 1 h at room temperature. After washing, the membranes were incubated for 1 h with horseradish peroxidase (HRP)-conjugated anti-swine IgG antibody. Immunoreactions were revealed using ECL (Amersham, USA). Positive plaques were rescreened twice until single positive clones were obtained.

### DNA sequencing

The positive clones were excised into phagemids according to the manufacturer’s instructions (Stratagene, USA). The phagemid DNAs were extracted and sequenced using vector primers (T7, T3 promoter). The sequences of positive clones were compared with existing sequences in GenBank by BLAST search (http://blast.ncbi.nlm.nih.gov/Blast.cgi).

### Identification of the full length cDNA sequence of *Ts*-ES-1 by 5’-RACE

The full-length *Ts*-ES-1 cDNA was obtained by 5’-RACE PCR from adult *T*. *spiralis* cDNA using a 5’-Full RACE kit (Takara, Japan), according to the manufacturer's instructions using *Ts-*ES-1 gene-specific primer GSP1: 5’-CCATTCAATTTTGCGTCACA-3’ and GSP2: 5’-CTTGCACAGCAACGTTGCAT-3’.

### Expression and purification of recombinant *Ts*-ES-1 protein (r*Ts*-ES-1)

The DNA encoding the full-length *Ts*-ES-1 without the signal peptide (22–172 amino acids) was amplified from *T*. *spiralis* adult total cDNA by PCR with the forward primer (5’-CGGGATCCgcgaaatcactggatgccgt-3’) and the reverse primer (5’-cgGAATTCctgtaatccattcaattttg-3’), then subcloned into the pET-28b (+) expression vector (Novagen) using the BamHI and EcoRI sites. After being transformed into *Escherichia coli* BL21 (DE3) cells, the expression of r*Ts*-ES-1 with a His-tag expressed at the N- and C-termini was induced with IPTG at a final concentration of 1 mM at 37°C for 4 h. After ultrasonic decomposition, the fractions of the induced cells were collected and analyzed by SDS-PAGE. The r*Ts*-ES-1 was expressed as an insoluble protein in inclusion bodies, then solubilized with 8 M urea and purified by Ni-affinity chromatography. The urea-solubilized r*Ts*-ES-1 was then refolded using a protein refolding kit (Novagen, Germany) according to the manufacturer’s instructions. The concentration of the r*Ts*-ES-1 was measured by the BCA method.

### Generation of anti-r*Ts*-ES-1 antibody

Antiserum against r*Ts*-ES-1 was produced in mice immunized subcutaneously with 25 μg of purified r*Ts*-ES-1 emulsified with an equal volume of the adjuvant ISA50v2 (Seppic, France), followed by two boost immunizations at 2-week intervals. One week after the last immunization, the mice were bled and the sera were collected and stored at -20°C.

### Western blot analysis

Protein samples including the crude somatic extracts of adult worm and muscle larvae, AES, MES, and r*Ts*-ES-1 were separated by SDS-PAGE with 12% polyacrylamide gel, then transferred onto an NC membrane (Millipore, USA). Another ES protein Ts87 was used as a loading control [18]. After being blocked with 5% (w/v) skimmed milk in PBS, the membrane was incubated with different *T*. *spiralis*-infected sera from swine (1:200), rabbit (1:500), mice (1:100) and human patients (1:200) or with mouse anti-r*Ts*-ES-1 sera (1:10,000). The corresponding IRDye 800CW-conjugated secondary antibody was used to detect specific antibody binding and visualized with the Odyssey CLx Infrared Imaging System.

### Real-time Quantitative PCR analysis of *Ts*-ES-1 gene transcription

To analyze the transcription of the *Ts-*ES-1 gene in different developmental stages of *T*. *spiralis*, total RNA was extracted from ML and adult worms with an RNA simple Total RNA Kit (TIANGEN, China) according to the manufacturer’s instructions. The cDNAs templates were reverse-transcribed from the same amount of total mRNA of ML and adult worms with an oligo dT primer using a Sensiscript Reverse Transcription Kit (Qiagen, Germany). A housekeeping *Trichinella* gene (GAPDH) was used as an internal control. Primers for detection of the *Ts*-ES-1 gene were designed as follows: 5’-gcgaaatcactggatgccgt-3’ (forward) and 5’-CTTGCACAGCAACGTTGCAT-3’ (reverse). Primers for GAPDH were 5’-TGCTTCTTGCACTACCAATGGCTTAG-3’ (forward) and 5’-ACCAGATGGACCATCGACTGTCTTTT-3’ (reverse). Real-time quantitative PCR was performed to determine gene transcription levels in ML and adult worms by using SYBR Premix Ex Taq (TaKaRa, Dalian China) in the DNA Engine Opticon 2 system (MJ Research, USA). All data were analyzed using the Opticon Monitor software, and threshold cycle (Ct) was calculated using the 2^−ΔΔCt^ method. After being normalized by GAPDH, the fold-change of the Ts-ES-1 gene expression level in adult worms was calculated relative to that in ML.

### Immunofluorescence assay (IFA)

*T*. *spiralis* muscle larvae collected from infected mice were fixed with 3% (v/v) paraformaldehyde and longitudinal sections were cut and Paraffin-embedded. After being blocked with normal goat serum for 1 h, the sections were incubated with the anti-r*Ts*-ES-1 mouse sera (1:100) for 2 h. Dylight 488-conjugated goat-anti-mouse IgG was used as the secondary antibody at a dilution of 1:100 for 1 h. A larval section incubated with sera from normal mice under the same conditions served as a negative control. These sections were washed three times with PBS and then examined under a fluorescence microscope (Leica, Germany).

### Immunization and challenge experiments

BALB/c mice were divided into three groups with 24 animals each. The mice in the first group were each vaccinated subcutaneously with 25 μg of r*Ts*-ES-1 emulsified with ISA50v2, then boosted twice using the same method at intervals of 2 weeks. The second and third groups were inoculated with ISA50v2 emulsified with PBS alone, or with PBS only, as controls using the same immunization regimen as the first group. Four mice from each group were sacrificed one week after each vaccination, and the sera and spleens were collected for immunological tests. Two weeks after the final boost, the remaining 12 mice in each group were each challenged orally with 500 infective *T*. *spiralis* muscle larvae. At 5 dpi, six mice from each group were sacrificed to collect adult worms to evaluate the adult worm reduction. The muscle larvae were examined for another 6 remaining mice from each group using a routine digestion method (described previously) at 45 dpi. The reduction in the adult worm or muscle larvae burden was calculated compared with the worms collected from the PBS control group.

### ELISA measurement of the antibody response

Mice sera were collected one week after each vaccination and measured for r*Ts*-ES-1-specific IgG, IgG1, and IgG2a antibodies by using an indirect enzyme-linked immunosorbence assay (ELISA). Briefly, flat-bottom, 96-well microtiter plates were coated overnight at 4°C with 100 μl r*Ts*-ES-1 at a concentration of 1.0 μg/ml in bicarbonate buffer (pH 9.6). After three washes with PBST, the microplates were blocked with 1% bovine serum albumin (BSA) in 100 μl of PBS for 1 h at 37°C. After another three washes with PBST, the microplates were probed with serial dilutions of immune sera for 1 h at 37°C. The plates were then washed and incubated with HRP-conjugated goat anti-mouse IgG, IgG1, or IgG2a for 1 h at 37°C. After the final wash, the substrate 3,3’,5,5’-tetramethylbenzidine (TMB) (BD, USA) was added to each well, and the reactions were stopped with 2 M H2SO4. Quantification of the reactions was determined by measuring the absorbance at 450 nm with an ELISA reader.

### Cytokine analysis

One week after each immunization, four mice from each group were sacrificed. The spleens were ground through a sterile steel mesh into lymphocyte separation medium. After being centrifuged, the spleen cells were resuspended in complete RPMI-1640 containing 10% FBS, 100 U/ml penicillin and 100 μg/ml streptomycin and adjusted to 1×10^7^ cells/ml. For *in vitro* stimulation, a total of 1×10^6^ splenocytes were incubated with 2 μg of r*Ts-*ES-1 in 200 μl of complete RPMI-1640 in 96-well flat-bottomed cell culture plates for ELISPOT. After cell stimulation for 48 h at 37°C in a humidified atmosphere containing 5% CO_2_, the cytokines IFN-γ, IL-2, IL-4 and IL-5 were detected using an ELISPOT kit (BD, USA), according to the manufacturer's instructions.

### Statistical analysis

All data were compared by analysis of variance (one-way ANOVA) and Student’s *t*-test using SPSS 15.0 software. The data were expressed as the means ± standard error. *p*<0.05 was regarded as statistically significant.

## Results

### Cloning of the cDNA encoding *Ts*-ES-1

**Fig 1:**
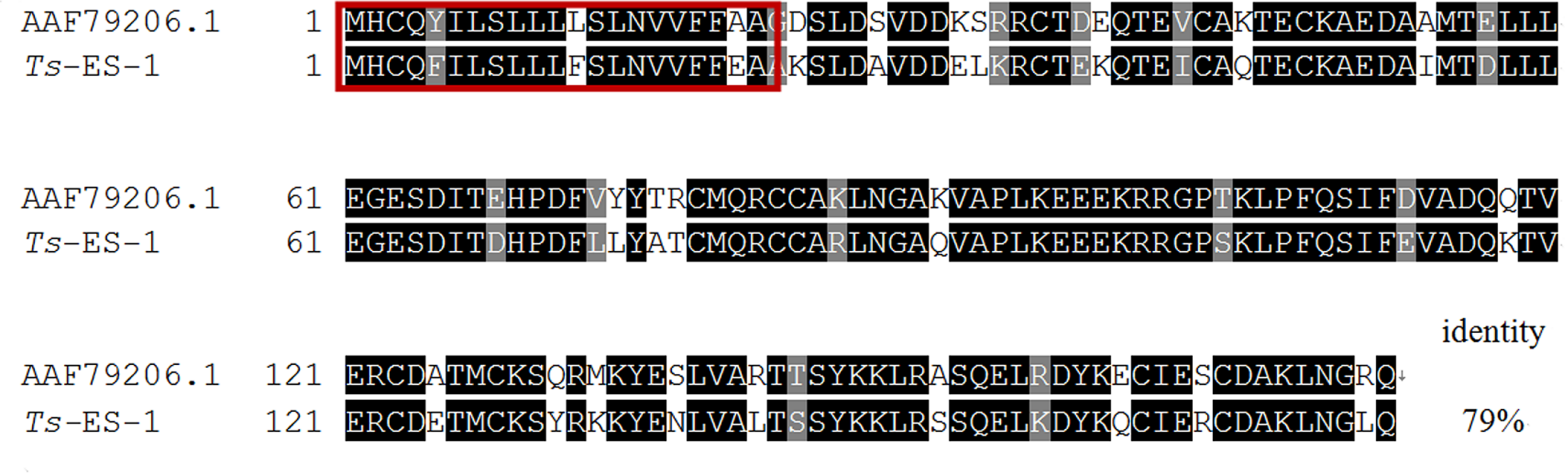
Alignment of the deduced amino acids sequence of *Ts-*ES-1 with a homologue from *T*. *pseudospiralis* (GenBank accession no. AAF79206.1). Sequences were aligned using CLUSTALW and prepared for display using BOXSHADE. Identical amino acids are shaded in black and similar amino acids in gray. The signal peptide in the first 21 amino acids is marked with a red rectangle. The percentage of sequence identity to the *T*. *pseudospiralis* homologue is shown at the end of the*Ts-*ES-1 sequence.

A *T*. *spiralis* adult cDNA library was immunoscreened with sera from swine experimentally infected with *T*. *spiralis* and a total of 43 positive clones encoding for 28 proteins were obtained (Table 1). Except for 8 enzymes involved in the intracellular processing, most of proteins identified are hypothetical *Trichinella* proteins with unknown function. Five of them contain signal peptide, 8 proteins contain apparent transmembrane domain, indicating these proteins could be the secreted proteins or surface antigens. Two of the positive clones strongly recognized by *T*. *spiralis*-infected swine sera encode the same open reading frame of a protein that shares 79% amino acid sequence identity with the 21 kDa excretory-secretory protein of *T*. *pseudospirali*s [19], hereby designated as *Ts*-ES-1 (excretory-secretory protein-1 of *T*. *spiralis*). The full-length DNA sequence of *Ts*-ES-1 contains 516-bp nucleotides that encode a protein of 172 amino acids, with the first 21 amino acids as a signal peptide (Fig 1). The molecular weight of full-length *Ts*-ES-1 is predicted to be 19.7 kDa, with a theoretical isoelectric point of 5.36.

**Table 1:** Identification of *T*. *spiralis* adult worm antigens recognized by pig infected sera.

Clone#	Name	NCBI ID	MW(kDa)	SP*	TM**
1-1-1	26S protease regulatory subunit 6B	XP_003371578.1	31.6	Yes	No
1-1-16	hypothetical protein Tsp_04117	XP_003374930.1	20.06	No	No
1-1-17	hypothetical protein Tsp_04117	XP_003374930.1	20.06	No	No
3-2-1	40S ribosomal protein S6	XP_003377362.1	57.29	No	No
3-2-3	putative fibronectin type III domain protein	XP_003374214.1	154.58	Yes	Yes
3-6-2	putative transcription initiation factor TFIID subunit 5	XP_003376268.1	106.06	No	Yes
4-1-1	heparan sulfate glucosamine 3-O-sulfotransferase 3A1	XP_003369975.1	39.93	No	No
5-1-1	sodium/potassium-transporting ATPase subunit beta-1-interacting protein 3	XP_003372552.1	81.04	No	Yes
6-1-6	Pre-rRNA-processing protein TSR1-like protein	XP_003379828.1	87.08	No	No
6-2-1	aspartate—tRNA ligase	XP_003373549.1	55.52	No	No
6-2-6	aspartate—tRNA ligase	XP_003373549.1	55.52	No	No
6-2-9	UDP N acetylglucosamine peptide	CDW56646.1	120.67	No	No
6-2-13	aspartate—tRNA ligase	XP_003373549.1	55.52	No	No
6-2-21	putative CBS domain pair	XP_003377393.1	128.1	No	Yes
6-2-23	UDP N acetylglucosamine peptide	CDW56646.1	120.67	No	No
7-2-3	40S ribosomal protein S25	XP_003370909.1	11.36	No	No
7-2-5	putative regulator	XP_003379635.1	28.29	No	Yes
7-2-10	conserved hypothetical protein	XP_003372742.1	93.77	No	Yes
7-2-14	putative regulator	XP_003379635.1	28.29	No	Yes
7-2-15	putative ATP synthase F1 delta subunit	AET09707.1	24.75	No	No
7-2-19	40S ribosomal protein S25	XP_003370909.1	11.36	No	No
7-2-23	putative ATP synthase F1 delta subunit	AET09707.1	24.75	No	No
8-3-1	5'-nucleotidase	XP_003374564.1	52.92	No	No
8-3-2	conserved hypothetical protein	XP_003374833.1	155.86	No	No
8-3-4	hypothetical protein	CBX25710.1	19.73	Yes	No
8-3-6	conserved hypothetical protein	XP_003374833.1	155.86	No	No
8-3-8	hypothetical protein	CBX25710.1	19.73	Yes	no
8-3-11	conserved hypothetical protein	XP_003382240.1	102.19	No	No
8-3-13	conserved hypothetical protein	XP_003374833.1	155.86	No	No
9-1-1	lonCoA ligase 5	XP_003380550.1	84.07	No	Yes
10-1-2	adult-specific DNase II-5	AAY32320.1	38.16`	Yes	No
10-1-3	adult-specific DNase II-5	AAY32320.1	38.16	Yes	No
10-1-8	60S ribosomal protein L19	XP_003374837.1	26.16	Yes	No
10-1-11	adult-specific DNase II-5	AAY32320.1	38.16	Yes	No
10-1-12	adult-specific DNase II-5	AAY32320.1	38.16	Yes	No
10-1-16	THO complex subunit 4	XP_003376451.1	21.71	No	No
10-2-1	hypothetical protein Tsp_00979	XP_003376752.1	26.07	No	No
10-2-2	hypothetical protein Tsp_00979	XP_003376752.1	26.07	No	No
10-2-5	hypothetical protein Tsp_00979	XP_003376752.1	26.07	No	No
10-2-8	putative tetratricopeptide repeat-containing domain protein	XP_003377351.1	118.64	No	No
10-2-14	actin-5C	XP_003373575.1	41.84	No	No
10-5-1	choline-phosphate cytidylyltransferase B	XP_003374840.1	80.6	No	No
10-5-5	hypothetical protein Tsp_12193	XP_003380508.1	34.46	No	Yes

*SP: signal peptide

**TM: transmembrane domain.

### Recombinant *Ts*-ES-1 (r*Ts*-ES-1) expression and recognition by *T*. *spiralis*-infected sera

**Fig 2:**
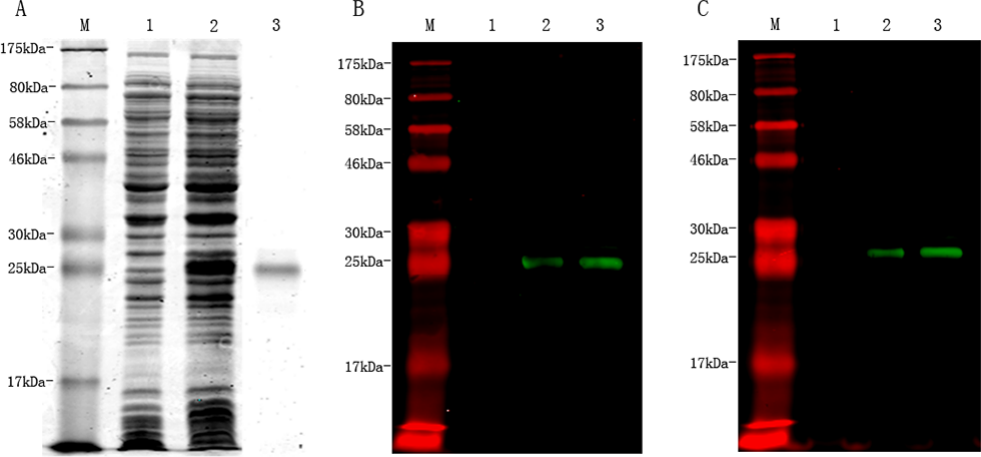
The expression and purification of r*Ts*-ES-1. SDS—PAGE analysis showing r*Ts*-ES-1 was highly expressed in IPTG-induced *E*. *coli* BL21 lysates (10 μl, Lane 2), not in the uninduced *E*. *coli* lysate(10 μl, Lane 1); the IMAC purified r*Ts*-ES-1 was loaded in Lane 3 (1 μg) (A). Western blot analysis showing the specific recognition of expressed r*Ts*-ES-1 in induced lysate (1 μl, Lane 2) or purified r*Ts*-ES-1 (500 ng, Lane 3), but not in uninduced *E*. *coli* lysates (1 μl, Lane 1), by mouse anti-*Ts*-ES-1 sera (1:10,000) (B) or by mouse anti-His monoclonal antibody (1: 5,000) (C).

Recombinant *Ts-*ES-1 (r*Ts*-ES-1) without the signal peptide (approximately 17 kDa) but with His tags at both the N-terminus and the C-terminus was expressed in *E*. *coli* BL21 (DE3) cells as an insoluble inclusion body. After being solubilized with 8 M urea, r*Ts-*ES-1 was purified with Ni-affinity chromatography and then refolded in 20 mM Tris, pH8.5. The molecular mass of the r*Ts*-ES-1 (with His tags) was approximately 24 kDa (Fig 2A), consistent with the calculated molecular weight including the His tags. Western blotting confirmed that the expressed r*Ts*-ES-1 could be recognized by mouse anti-*Ts*-ES-1 sera (Fig 2B) and anti-His antibody as well (Fig 2C).

**Fig 3:**
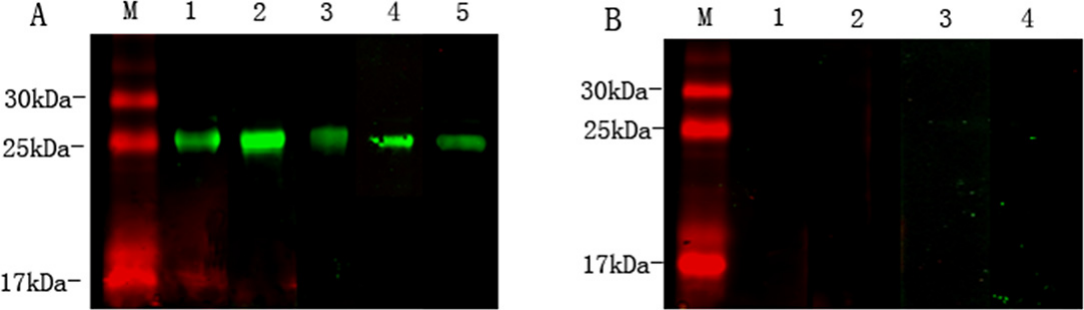
Recognition of recombinant *Ts*-ES-1 by anti-*Ts*-ES-1 antisera. A. Western blot analysis showing the recognition of recombinant *Ts-*ES-1 (500 ng) by sera from *T*. *spiralis*-infected mice (Lane 1), swine (Lane 2), rabbits (Lane 3), human patients (Lane 4) and mouse anti-*Ts*-ES-1 sera (Lane 5). B. Western blot showing there is no recognition of the same amount of *Ts*-ES-1 (500 ng) by sera from normal mice (Lane 1), normal swine (Lane 2), normal rabbits (Lane 3) and healthy human (Lane 4).

The purified r*Ts*-ES-1 was used to evaluate its antigenicity by immunoblotting with different *T*. *spiralis*-infected animal or human sera. The results demonstrated that r*Ts*-ES-1 was recognized not only by mouse anti-r*Ts*-ES-1 antisera, but also by all *T*. *spiralis*-infected animal sera from swine, rabbits, mice, and human patients with trichinellosis (Fig 3A), whereas no reaction was detected with sera from healthy people or normal animals (Fig 3B). The results indicate that *Ts*-ES-1 is a highly immunogenic antigen and induces a strong antibody response in hosts during natural infection.

### Stages of *Ts*-ES-1 expression

**Fig 4:**
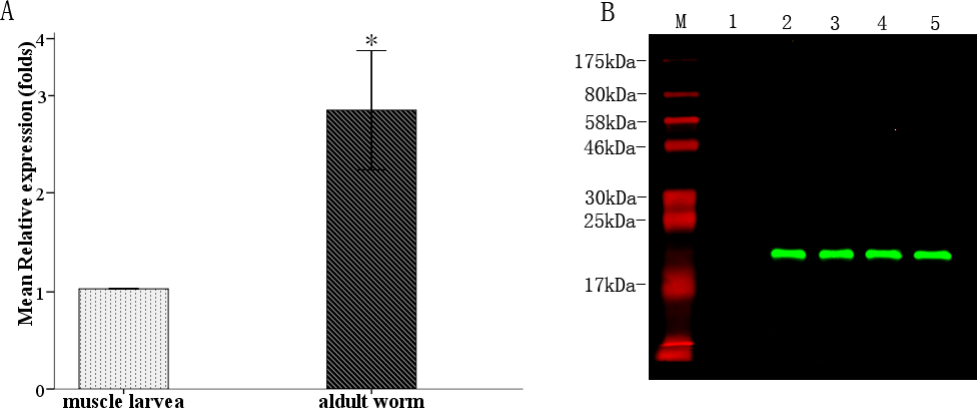
Stage expression of *Ts-*ES-1 expression in *T*. *spiralis*. A. Real-time quantitative PCR analysis of stage expression levels of *Ts-es-1* gene. After being normalized with GAPDH, the fold-change of the *Ts-es-1* gene expression level in adult worms was calculated relative to that in ML. The fold values are presented as the mean of three experiments ± standard deviation. The asterisk (*) indicate significant difference (*p* < 0.05) between the transcription of *Ts-es-1* gene in adult worm and in ML. B. Western blot analysis showing the native *Ts-*ES-1 protein recognized by mouse anti-*Ts-*ES-1 antisera in the somatic extracts of ML (Lane 2); somatic extracts of adult worms (Lane 3); ES products of ML (Lane 4) and ES products of adult worms (Lane 5). There was no reaction of mouse anti-*Ts-*ES-1 antisera to recombinant Ts-87 (Lane 1), another ES protein of *T*. *spiralis*.

Real-time quantitative PCR was performed to observe the transcription level of *Ts*-ES-1 gene at ML and adult worm life stages of *T*. *spiralis*. As shown in Fig 4A, After being normalized by GAPDH, the fold-change of the *Ts*-ES-1 gene expression level in adult worms was calculated relative to that in ML. We found that the gene expression of *Ts*-ES-1 increased significantly to 2.8-fold in the adult stage (p<0.05).

The protein expression level and distribution of native *Ts-*ES-1 was determined by Western blot with anti-*Ts*-ES-1 antisera raised in mice through immunization with r*Ts*-ES-1. The results demonstrated that a tight band at approximately 20 kDa was detected by anti-*Ts*-ES-1 antisera not only in somatic extracts but also in ES products of both *T*. *spiralis* adult and muscle larval worms (Fig 4B), indicating that the native *Ts*-ES-1 is a secreted protein in both muscle larvae and adult stages. It is consistent with the finding that the same protein was identified in the pre-mature adult worm at 20 hours post-infection [20], and the homologue in *T*. *pseudospiralis* was found in the excretory-secretory proteins of muscle larvae [19]. The actual molecular mass (20 kDa) is greater than that predicted by the sequence for the mature protein without signal peptide (17 kDa) possibly due to post-translational modification of the natural protein. The mouse anti-*Ts*-ES-1 sera didn’t recognize recombinant Ts87, another ES protein of *T*. *spiralis* as a loading control [18].

### Immunolocalization of *Ts*-ES-1

**Fig 5:**
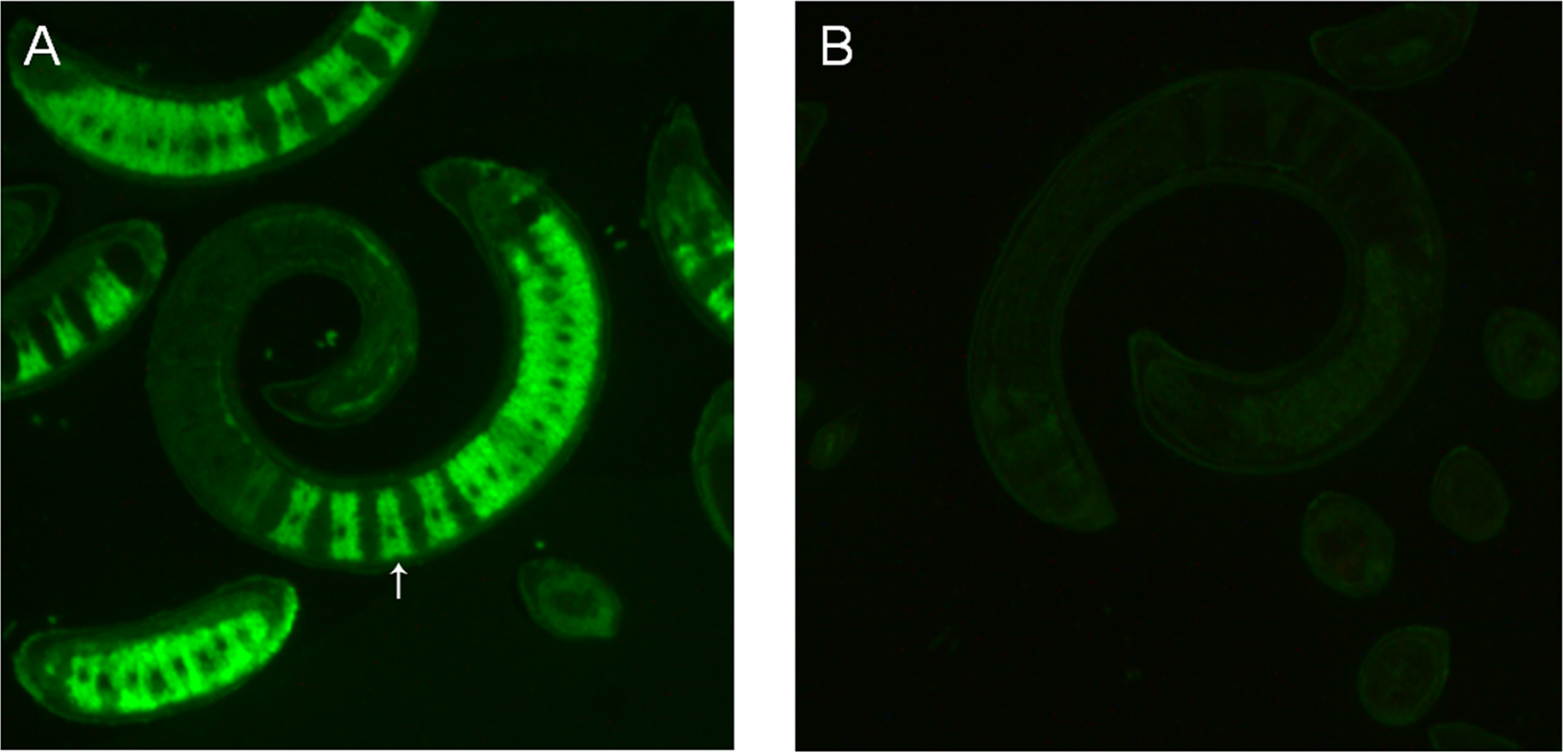
Immunolocalization of *Ts*-ES-1 in sections of *T*. *spiralis* muscle larvae (400×). Fluorescence was detected with Dylight 488-conjugated anti-mouse IgG secondary antibody. Longitudinal sections of *T*. *spiralis* ML were probed with mouse anti-*Ts-*ES-1 antisera (1:100) and native *Ts-*ES-1 was shown to be located intensively in the stichocytes at the posterior portion of *T*. *spiralis* ML stichosome (A). Nothing was detected when normal mouse serum at the same dilution was used as control (B).

To localize the native *Ts*-ES-1 in the parasites, sections of *T*. *spiralis* muscle larvae were allowed to react with mouse anti-r*Ts*-ES-1 sera and then incubated with Dylight488-conjugated goat-anti-mouse IgG. The results of IFA revealed that the native *Ts*-ES-1 was strongly and exclusively distributed in the stichocytes of *T*. *spiralis* muscle larvae stichosomes. In contrast, little reactivity was detected in sections of the muscle larvae when probed with normal mouse sera (Fig 5). The results clearly reveal that native *Ts*-ES-1 is highly expressed in stichocytes, consistent with the Western blot results showing that *Ts-*ES-1 is secreted and present in the ES products of larval and adult worms (Fig 4B).

### Immunogenicity of r*Ts*-ES-1

**Fig 6:**
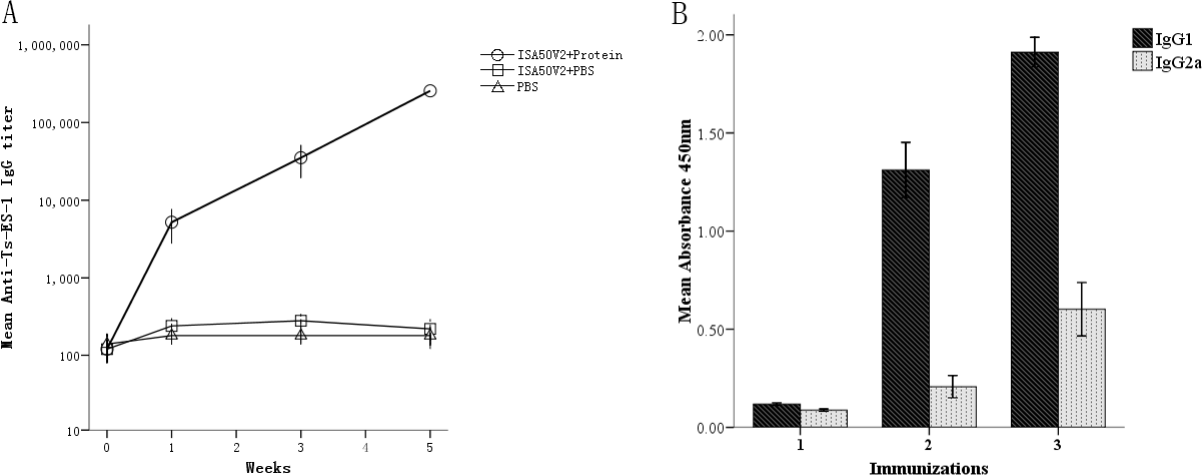
Antibody responses of vaccinated mice against r*Ts-*ES-1 formulated with ISA50v2 adjuvant before challenge with *T*. *spiralis* ML. A. Specific IgG titer was detected one week after each immunization. B. Serum IgG subclass responses (OD at 1:100 dilutions) in mice upon vaccinations with r*Ts*-ES-1 formulated with ISA50v2 were detected one week after each immunization. The values are presented as the arithmetic mean of four mice in the r*Ts*-ES-1 group ± standard error.

Recombinant *Ts*-ES-1 was used to immunize mice three times, and the mouse serum samples were collected from each immunized mouse one week after each immunization. The antibody titers of the serum samples against r*Ts*-ES-1 were measured using ELISA. A high titer of specific IgG antibodies was elicited in all of the immunized mice one week after each immunization, and the highest IgG titer reached 1:256,000 after the third immunization (Fig 6A). The IgG subclass antibody levels were measured to further assess the efficacy of r*Ts*-ES-1 in induction of the different IgG subclass responses *in vivo*. The results demonstrated that the predominant IgG subclass was IgG1, but there was also a significant level of IgG2a response especially after the first immunization boost (Fig 6B).

**Fig 7:**
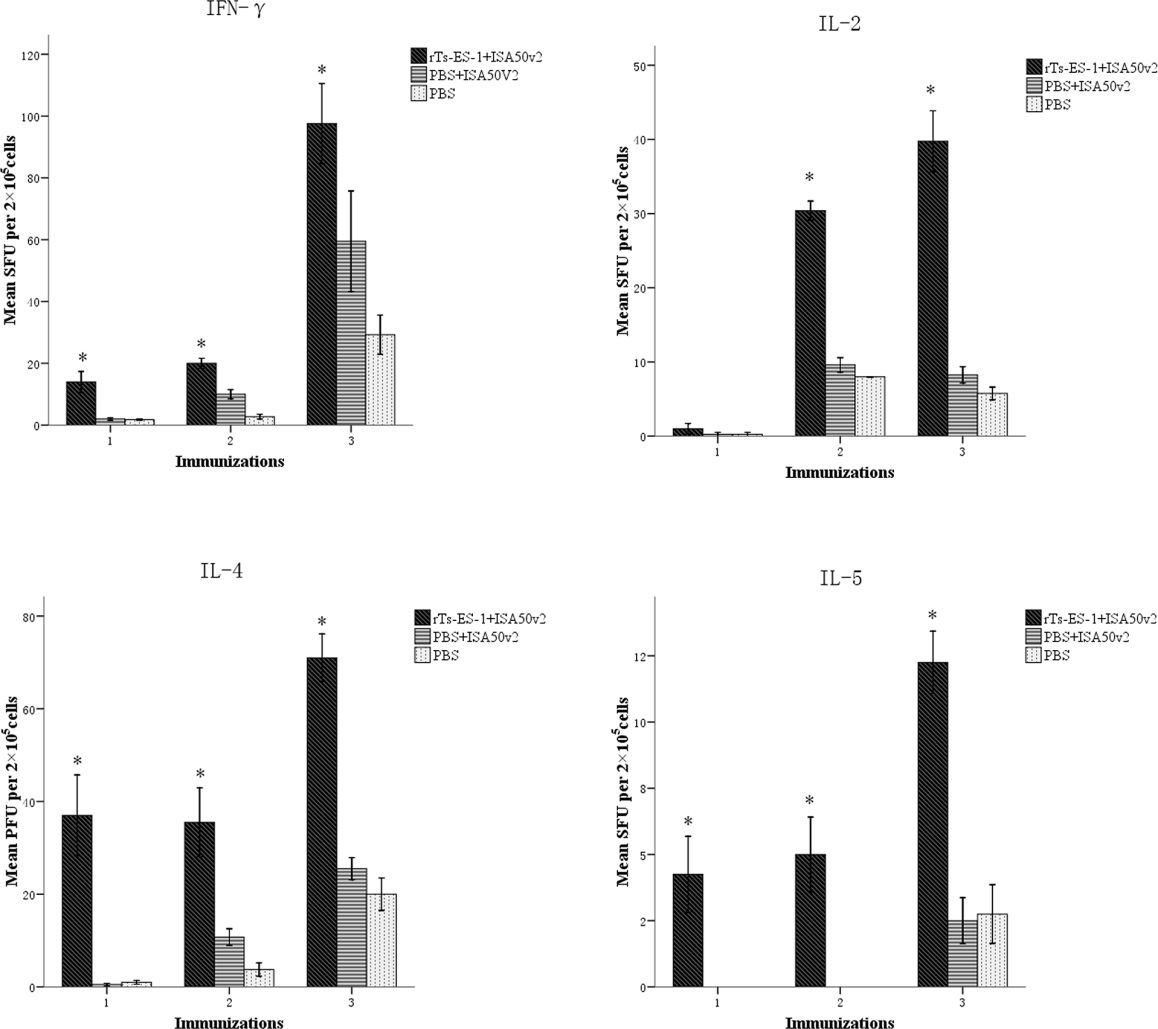
Cytokines secreted by splenocytes from BALB/c mice immunized with r*Ts*-ES-1 upon stimulation with 2 μg of r*Ts-*ES-1 *in vitro* before challenge with *T*. *spiralis* ML. Splenocytes secreting IFN-γ, IL-2, IL-4 and IL-5 were detected by ELISPOT one week after each immunization. The values are presented as the arithmetic mean of four mice in each group ± standard error. The asterisks (*) indicate significant differences (*p* < 0.01) between the r*Ts*-ES-1 and control groups.

The cytokines secreted by immunized mouse splenocytes upon stimulation of r*Ts*-ES-1 *in vitro*, including IFN-γ, IL-2, IL-4, and IL-5, were measured using ELISPOT. The levels of the typical Th1 cytokines (IFN-γ, IL-2) and Th2 cytokines (IL-4, IL-5) were significantly elevated in mice vaccinated with r*Ts*-ES-1 compared to the adjuvant-alone control group (Fig 7), and the Th2 cytokines IL-4 and IL-5 were significantly increased even after the first immunization. Our results showed that r*Ts*-ES-1 vaccination induced mixed Th1 and Th2 responses in mice in terms of the antibody response and cytokine production.

### Partially protective immunity elicited by r*Ts*-ES-1

Partially protective immunity against *T*. *spiralis* infection induced by r*Ts*-ES-1 was observed in immunized BALB/c mice. The result of the challenge experiment showed that mice immunized with r*Ts*-ES-1 formulated with the adjuvant ISA50v2 for three times, then challenged with 500 *T*. *spiralis* infective larvae, induced a 27% adult worm reduction (138 ± 20) and 42.1% muscle larvae (ML) reduction (3782 ± 766), which was significantly different from adjuvant-alone control group (adult 197 ± 20; ML 6780 ± 938) (*p*<0.01) (Table 2). There was no significant difference in the adult worm and muscle larvae burden between the adjuvant-alone (ISA50v2) and PBS-alone control groups. These results show that *T*. *spiralis*-secreted *Ts*-ES-1 enable the induction of partial protective immunity against *T*. *spiralis* infection in mice.

**Table 2:** Protective immunity elicited by immunizing r*Ts*-ES-1 in mice against challenge with 500 *T*. *spiralis* larvae.

Group	Mean adult worm burden ± SD (mouse#)	adult worm reduction	Mean muscle larvae per gram muscle ± SD (mouse#)	Muscle larvae burden reduction
PBS	189 ± 19 (6)		6532 ± 618 (6)	
ISA50V2+PBS	197 ± 20 (6)		6780 ± 938 (6)	
ISA50V2+ rTs-ES-1	138 ± 20 (6)	27% *	3782 ± 766 (6)	42.1% *

*p<0.01 compared with adjuvant control group.

## Discussion

Generally, the lack of an effective vaccine and a reliable early diagnostic method for *Trichinella* infection gives rise to the establishment of mature and encapsulated *Trichinella* muscle larvae, which are usually resistant to treatment with anthelmintic drugs. With the purpose of discovering an effective vaccine or diagnostic antigen, the adult cDNA library of *T*. *spiralis* was immunoscreened with sera from pigs experimentally infected with 20,000 *T*. *spiralis* muscle larvae.

Infection with the *Trichinella* parasite results in the induction of immunity in hosts that is strong enough to trigger rapid defense against secondary infection [11–13,21]. The resistance to a secondary infection in pigs was infection dose-dependent. Infection with 25,000 muscle larvae induced almost complete resistance to re-infection [22]. In addition to activated mast cells [23], eosinophils [24], mucosal immunity [25] and Th1 cellular responses [26], which were determined to be involved in protective immunity against *Trichinella* infections, the most effective and consistent protective effects are attributed to specific anti-*Trichinella* antibodies [27]. Passive transfer of infected immune serum or parasite-specific monoclonal antibodies against a parasite-specific glycoprotein confers effective immunity on naive pups against *Trichinella* infections [21,28]. In this study, the sera or antibodies collected from protected pigs, which are elicited by infection with a high dose of infective muscle larvae (20,000), are supposed to recognize the antigens of *T*. *spiralis* that induce protective immunity.

Total 43 positive clones encoding for 28 proteins were identified by immunoscreening the adult cDNA expression library with *T*. *spiralis*-infected swine sera. One of the clones was identified as a *Trichinella*-secreted protein sharing 79% amino acid sequence identity with a previously identified secreted protein in *T*. *pseudospiralis* and was therefore designated as *Ts*-ES-1. There is no any homologue or functional domain found in other nematodes or species except for genetically related *T*. *pseudospiralis*, therefore, it is a *Trichinella*-specific protein with unknown function. *Ts-*ES-1 contains 172 amino acids with a typical signal peptide in the first 21 amino acids. The expression of *Ts-*ES-1 was detected in both the adult and the muscle larval stages of *T*. *spiralis* at the mRNA and protein levels. These results are consistent with the detection of the native *Ts-*ES-1 protein in the ES products of both the adult and the muscle larval worms identified in this study. Furthermore, secreted *Ts-*ES-1 was localized in the stichocytes of the muscle larvae using an IFA with specific antibodies. Similar to *Trichuris* nematodes, *Trichinella* stichocytes are glandular unicellular cells arranged in a row along the posterior portion of the esophagus that produce and secrete proteins into the lumen of the esophagus with different biological functions [29,30]. Nematode-released ES products are the primary interface between the parasites and the hosts, playing a wide range of roles crucial for their survival and reproduction. It has been determined that the proteins in ES products are essential for invading into host tissues [31], feeding [32], reproduction [33] and modulating the host immune system to evade host immune attack [34,35]. Therefore, nematode-secreted proteins have been targeted as major vaccine candidates [36]. Vaccines based on ES products have been reported for many parasites [15,37–39]. An ES product of adult *Brugia malayi*, Bm-iPGM, could induce 58.2% protection against larval challenge in BALB/c mice and 65–68% protection in *M*. *coucha* [40]. The 52.8-kDa protein from the ES products of *S*. *japonicum* could induce a 35.32% or 26.19% reduction in the worm burden and a 33.17% or 31.7% lower liver egg count in two experiments in vaccinated mice [41]. The ES antigens of *T*. *spiralis* are directly exposed to the host's immune system and are the main antigens that induce the immune responses in the host [42]. It has been reported that the ES products of *T*. *spiralis* could provide protective immunity [15], and some of the proteins identified within the ES products of *T*. *spiralis* have achieved some success in vaccines or immunodiagnosis [43,44]. For these reasons, we have further explored the *Trichinella*-secreted *Ts*-ES-1identified in this study for its potential as a vaccine against *T*. *spiralis* infection.

In this study, mice vaccinated with r*Ts*-ES-1 formulated with ISA50v2 adjuvant exhibited a significant reduction in the adult worms (27%, 138 ± 20 vs 197 ± 20) and muscle larvae (42.1%, 3782 ± 766 vs 6780 ± 938) after being challenged with *T*. *spiralis* compared to the adjuvant control group (*p*<0.01). The r*Ts*-ES-1-induced protection was associated with a high level of anti-*Ts*-ES-1 antibody, including increased total IgG and the IgG1 and IgG2a subtypes, and the production of the splenocyte-secreted cytokines IFN-γ, IL-2, IL-4 and IL-5. It is commonly believed that the Th2 immune response is essential for protective immunity for helminth infections [45–47]. The humoral response contributes greatly to resistance against *Trichinella* infection by entrapping and expulsing infective larvae, reducing the fecundity of adult worms and eliminating newborn larvae [26]. In addition, it has also been demonstrated that the combined humoral and cellular immune responses are important for immunity against *T*. *spiralis* infection [48]. Some experimental results suggest that the cellular response as well as the humoral response may be involved in the mechanisms of protective immunity induced by recombinant protein immunization [49–51]. In this study, mice immunized with r*Ts*-ES-1 produced not only a major Th2-associated immune response (IgG1 antibody, IL-4, and IL-5) but also a Th1-like response evidenced by high titers of IgG2a antibody, IFN-γ and IL-2. The results indicated that mice vaccinated with r*Ts*-ES-1 produced a mixed humoral and cellular immune response that may contribute to the protective immunity observed in this study.

However, the worm reduction induced by immunization with r*Ts*-ES-1 against *T*. *spiralis* larval challenge in this study was not high (adult worms reduction 27%; muscle larvae reduction 42.1%), similar to the level induced by other *T*. *spiralis* vaccine candidates identified so far [9,18,50–55]. The non-sterilizing immunity or low protection is a dilemma not only for vaccine development against *Trichinella* infection, but also for all other helminth infections. WHO admitted that the goal with consistent induction of 40% protection or better for Schistosomiasis was not reached with any of antigens in clinical trials [56]. The less than 50% protection was also seen in hookworm vaccines currently in clinical trials [57]. The low protection induced by single vaccine immunization for helminth infection may be caused by the complexity of the life cycle, diversity of stage-specific antigens, immune-evasion strategies and the modulatory effect of host responses [52]. However, the disease development by helminthic parasites usually depends on the intensity of infection [57]. Therefore, reducing the worm burden by vaccination, even not sterilizing, may significantly reduce the manifestation and seriousness of disease [57]. Nevertheless, new strategies are needed to improve the protection of vaccine against *Trichinella* infection. These strategies may include the multivalent vaccine with combination of more than one vaccine antigens or protective epitopes [58], vaccine that induces intestinal local immunity [18]. Glycoproteins induced strong immune response during infection and antibody against a tyvelose motif on several secreted and surface glycoproteins in *T*. *spiralis* L1 larvae effectively prevented niche establishment of the parasites in intestine epithelia, therefore a good vaccine target [21,28,59].

All results described in this study demonstrate that the newly identified *Ts-*ES-1 secreted by *T*. *spiralis* stichocytes plays an important role in the survival of *T*. *spiralis* in its host and therefore is a potential candidate for vaccine development against trichinellosis. The specific function of this *Trichinella*-secreted protein and the enhancement of immunogenicity and vaccine efficacy induced by this antigen are under further investigation.
